# Multi-frequency spatial frequency domain imaging: a depth-resolved optical scattering model to isolate scattering contrast in thin layers of skin

**DOI:** 10.1117/1.JBO.29.4.046003

**Published:** 2024-04-22

**Authors:** Luigi Belcastro, Hanna Jonasson, Rolf B. Saager

**Affiliations:** Linköping University, Department of Biomedical Engineering, Linköping, Sweden

**Keywords:** spatial frequency domain imaging, wound healing, light transport models, depth reconstruction

## Abstract

**Significance:**

Current methods for wound healing assessment rely on visual inspection, which gives qualitative information. Optical methods allow for quantitative non-invasive measurements of optical properties relevant to wound healing.

**Aim:**

Spatial frequency domain imaging (SFDI) measures the absorption and reduced scattering coefficients of tissue. Typically, SFDI assumes homogeneous tissue; however, layered structures are present in skin. We evaluate a multi-frequency approach to process SFDI data that estimates depth-specific scattering over differing penetration depths.

**Approach:**

Multi-layer phantoms were manufactured to mimic wound healing scattering contrast in depth. An SFDI device imaged these phantoms and data were processed according to our multi-frequency approach. The depth sensitive data were then compared with a two-layer scattering model based on light fluence.

**Results:**

The measured scattering from the phantoms changed with spatial frequency as our two-layer model predicted. The performance of two δ-P1 models solutions for SFDI was consistently better than the standard diffusion approximation.

**Conclusions:**

We presented an approach to process SFDI data that returns depth-resolved scattering contrast. This method allows for the implementation of layered optical models that more accurately represent physiologic parameters in thin tissue structures as in wound healing.

## 1 Introduction

The skin is one of the largest organs of the human body, and it acts as an interface to the outside world and as a barrier to protect from external pathogens and other hazards. A wound is formed when skin is damaged or otherwise unable to perform its functions.^1^^,^^2^ The most common method used for evaluating the progression of the healing process is visual inspection by a trained physician. Parameters, such as wound size, color, granulation, and presence of secretions or necrotic tissue are annotated at different points in time and used to make an estimate of how much a wound has progressed.^3^^–^^5^ This method is fast, repeatable, and non-invasive, but the dependence on the expertise of the clinical personnel makes it a non-reliable way to obtain a consistent diagnosis.^6^ A more specific method to obtain information on the wound is biopsy, followed by staining and cell histology. This approach gives more structural information at the cellular level, but it is invasive, extremely localized and non-repeatable (especially on patients *in-vivo*), so it is only performed when there is already suspicion of malignant tissue. An ideal technique for wound assessment would combine the advantages of both visual inspection and histology, being non-invasive, repeatable in time, and specific, while presenting an objective measurement of parameters that can be employed as a metric for the evaluation of the efficiency of different regeneration treatments.

Optical techniques are non-invasive, fast methods for performing measurements on tissue using light. They satisfy most of the prerequisites that we want from an ideal wound healing assessment method: from the way that light interacts with tissue, it is possible to quantitatively measure concentrations of biological chromophores and indirectly obtain structural information on a microscopic scale, over a large field of view. Optical techniques are already widely utilized in clinical practice, where they are used for determining blood perfusion and blood oxygen saturation,^7^^–^^9^ detecting cancer,^10^^,^^11^ and optimizing photodynamic therapy,^12^^,^^13^ among other applications.^14^^,^^15^

Spatial frequency domain imaging (SFDI) is an optical imaging technique that makes use of structured light illumination to quantitatively measure the absorption ($\mu_{a}$) and reduced scattering ($\mu_{s}^{\prime }$) coefficients in tissue and separate their effects by making use of the frequency-specific response of the tissue.^16^ The absorption of light contains information about the concentration of molecules that are capable of absorbing photons (e.g., melanin, haemoglobin, etc.), whereas light scattering is dependent on the density and size distribution of the microscopic structures (e.g., collagen fibers, cell nuclei, etc.). These properties make SFDI a potentially valuable tool for the diagnosis of wound healing as it is able to measure objective parameters related to tissue function (e.g., blood concentration and oxygenation) and morphology (e.g., cellular proliferation and tissue remodeling) in a fast, non-invasive, and repeatable way. The technique, in its current state, has a few limitations, most notable of which is that the models of light transport, used to quantify the absorption and scattering parameters, presume a semi-infinite, optically homogeneous geometry. When looking at the practical application of wound assessment, it is evident that a homogeneous model is not an accurate representation of the underlaying physiology: biological tissue is quite heterogeneous and contains multiple thin layers with different properties. For the purpose of overcoming the limitations of homogeneous models we introduce a two-layer model composed of a thin layer, with a thickness in the range of hundreds of microns (which simulates the new tissue growth) and a thick layer, which can be approximated by a semi-infinite geometry and simulates the underlaying wound site. The introduction of the two-layer model alone, however, is not sufficient. To discriminate between the optical properties between the top and the bottom layer, we also need a dataset that contains information spanning multiple penetration depths of light into the tissue volume. It was shown in previous investigations that, when acquiring data over wavelengths of light spanning the visible and near infrared, each will have a different penetration depth depending on the optical properties of the tissue, with longer wavelengths (e.g., near-infrared) normally having longer penetration depths compared with shorter wavelengths (e.g., visible).^17^

However, in the context of wound healing, this approach is not as applicable because the absorption contrast between layers is less pronounced. Scattering contrast, on the other hand, would contain distinct information about depth-specific structural changes in the wound site. SFDI also has a unique property of allowing for varying the penetration depth of the light patterns independently from the wavelength of light. This is made possible by means of the spatial frequency ($f_{x}$) of the illumination patterns.^18^ Sinusoidal patterns with higher frequencies have a lower penetration depth compared with patterns with lower frequencies. By acquiring multiple datasets with an increasing average spatial frequency, we are effectively sampling smaller and smaller volumes, which contain information in increasing proportion from the superficial layer, compared with the bottom layer. This multi-frequency approach is particularly suited to detecting variations in scattering contrast over thin superficial layer, as absorption contrast is discernible mostly at low $f_{x}$ (and higher penetration depth), whereas scattering has a larger influence at high $f_{x}$ (smaller penetration depth).

In this study, we acquire an SFDI dataset at several spatial frequency and then subdivide it in multiple overlapping sub-sets containing increasing spatial frequencies. These sub-sets, containing data with different penetration depth by means of different $f_{x}$, constitute the basis of the two-layered model proposed in this study and are used to determine the layer specific scattering coefficients, which for the practical application of wound assessment can be correlated to new cell growth (re-epithelialization) and morphological changes within the underlying wound bed (remodeling). To test the validity of the model, SFDI data were acquired on silicone multi-layered optical phantoms with known properties and then processed according to the previously described multi-frequency approach. These tissue simulating multi-layer phantoms were designed to mimic the relative, layer-specific optical properties observed during re-epithelialization of a wound. Using the individual properties of the two layers of the phantoms and their geometrical parameters, we compared the experimental data with the proposed two-layer model using three different analytical models of light transport. The root mean square percentage error (RMSPE) of the modeled data was then calculated as an evaluation metric to determine the performance of the models in different ranges of parameters.

## 2 Two-layer Scattering Model

Thin layered models of skin, based on the difference between the absorption in the epidermis and dermis layers, are already used in clinical applications to account for the different absorption levels of epidermis and dermis.^17^^,^^19^ Scattering-based multi-layer models, however, are less common. SFDI can be used to measure both absorption and scattering in a tissue, but for the purpose of this study, we consider differences in the scattering properties between the layers. As mentioned, in the context of wound healing, the difference in absorption between the layers is less pronounced, whereas the scattering coefficient can be subjected to large variations that give insight on the changes in tissue morphology due to the healing process. A two-layer scattering model based on the spectral difference in depth penetrance between the layers was already developed by our group to investigate morphologic changes within skin pigmentation and melanoma; however, that approach was optimized to isolate tissue layer thicknesses of ∼0.2 to 4 mm.^20^ In the present work, we aim to mimic the physiology of a healing wound, in which sub-cellular organelles of different sizes and new epithelial cells give different sources of scattering-based contrast. The end goal is to be able to measure this scattering contrast between thin tissue layers (0.05 to 0.5 mm).

**Fig. 1:**
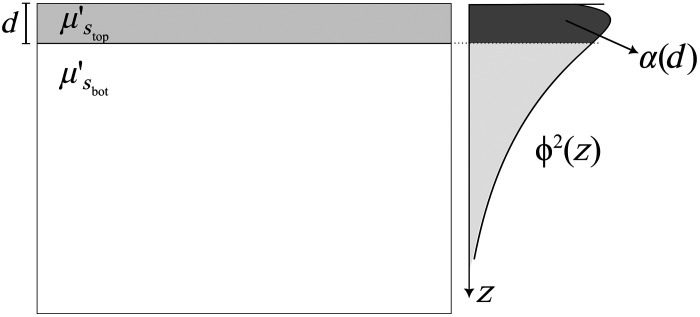
2D drawing of the geometry of the two-layer scattering model (left). A thin layer of thickness d with scattering coefficient $\mu_{S_{\mathrm{top}}}^{\prime }$ is laid on a semi-infinite layer with scattering coefficient $\mu_{S_{\mathrm{bot}}}^{\prime }$. Both layers are assumed to be homogeneous and extend infinitely in the xy plane. The light fluence ϕ (right) decreases exponentially from the surface in the z direction (depth). The weighting coefficient α(d) is defined as the partial contribution of fluence squared over the thin layer of thickness d. The contribution is obtained by integrating $\phi^{2}$ up to d and normalizing to the total integral of $\phi^{2}$.

The geometry of our two-layer model is represented in Fig. 1. The thin top layer has a finite thickness d and a scattering coefficient $\mu_{S_{\text{top}}}^{\prime }$. The bottom layer is a semi-infinite slab, with scattering coefficient $\mu_{S_{\mathrm{bot}}}^{\prime }$. The index of refraction (n) is also assumed to be the same in both layers to avoid unwanted reflections and refractions due to index mismatch.

There are three parameters in this scattering model: $\mu_{S_{\text{top}}}^{\prime }$, $\mu_{S_{\mathrm{bot}}}^{\prime }$, and d. When performing normal SFDI measurements, we obtain a single measured scattering coefficient ($\mu_{s_{\mathrm{meas}}}^{\prime }$), which is assumed to derive from a single, homogeneous, and semi-infinite geometry. We want to define a modeled scattering coefficient ($\mu_{s_{\mathrm{mod}}}^{\prime }$) that models the behavior of $\mu_{s_{\mathrm{meas}}}^{\prime }$ and takes into consideration the relative contributions of the three parameters of the two-layer model. A first assumption that we make is that $\mu_{s_{\mathrm{mod}}}^{\prime }$ is a linear combination of the two free scattering parameters, $\mu_{S_{\text{top}}}^{\prime }$ and $\mu_{S_{\mathrm{bot}}}^{\prime }$, given as

$$\mu_{S_{\mathrm{mod}}}^{\prime }=\alpha (d)\mu_{S_{\text{top}}}^{\prime }+(1-\alpha (d))\mu_{S_{\mathrm{bot}}}^{\prime },$$ (1)

where α(d) is a weighting coefficient representing the layer-specific partial volume contribution of scattering, which is dependent on the thickness of the thin layer (d).

### 2.1 Fluence-Based Partial Volume Contribution (α)

Next, we need to find a metric to model α(d) as the relative contribution to light scattering from the top layer. Symmetrically, (1−α(d)) represents the relative contribution to light scattering from the bottom layer. The metric that we chose to model α(d) is based on light fluence (ϕ). An advantage in using light fluence is that it can be calculated using different models that give an expression of fluence in three dimensions, so they contain the information about depth that we need to model light transport in multiple stacked layers. Because our technique uses planar illumination and wide-field imaging for detection, we assume the fluence to be constant relative to the field of view of a given pixel in the xy directions, simplifying the problem to one dimension in the z direction.

In our equation, we used the photon hitting density,^21^ which for our source–detector configuration corresponds to the fluence squared ($\phi^{2}$), because this is a co-located forward-adjoint model. Both our light source and detector (planar illumination and wide-field imaging, respectively) have the same probability distribution for a photon to reach a certain depth z (represented by ϕ(z)). When multiplying the two functions for the source and detector, we end up with the same function squared. Because we are interested in the relative photon contribution from each layer, we define α(d) as the integral of the fluence in depth over the top thin layer, normalized to the integral of the total fluence, given as

$$\alpha (d)=\frac{\int_{0}^{d}\phi^{2}(z)dz}{\int_{0}^{\infty }\phi^{2}(z)dz}.$$ (2)

### 2.2 Influence of Spatial Frequency ($f_{x}$)

To separate the optical properties of the two layers, we want to perform measurements that investigate different volumes of tissue. To do so, we make use of a specific property of SFDI: the penetration depth of the sinusoidal patterns is inversely proportional to their spatial frequency.^18^^,^^22^ This is possible because we are performing measurements of $\mu_{S_{\mathrm{meas}}}^{\prime }$ on a two-layered geometry, so changing the penetration depth of light will change the relative contribution of each layer to the overall scattering of light, as opposed to homogeneous models in which every measurement is supposed to return the same value, independently from the penetration depth of light. In Eq. (1), the layers with scattering coefficients $\mu_{S_{\mathrm{top}}}^{\prime }$ and $\mu_{S_{\mathrm{bot}}}^{\prime }$ are assumed to be homogeneous, so their values are independent from $f_{x}$. The parameter α, instead, is based on light fluence, so it is also dependent on the spatial frequency, in addition to the thickness of the thin layer (d); it is given as

$$\alpha (d,f_{x})=\frac{\int_{0}^{d}\phi^{2}(z,f_{x})dz}{\int_{0}^{\infty }\phi^{2}(z,f_{x})dz}.$$ (3)

### 2.3 Analytical Models of Fluence (ϕ)

The last part needed to define our two-layer model is to choose a suitable model of light fluence (ϕ) that solves the radiative transport equation, specific to the spatial frequency domain. There are several options available to calculate a statistically reliable representation of ϕ: in general, from analytical models that make use of approximations to obtain simplified mathematical formulations of ϕ^23^^–^^25^ to computer simulations using the Monte Carlo method that use realistic geometries and random propagation for millions of photons.^26^^,^^27^ In this study, we selected and evaluated the performance of two analytical models of light fluence. This decision was made because of the ease of implementation and much faster computation times compared with simulation-based models. In addition, normal Monte Carlo methods cannot currently perform direct simulations in the spatial frequency domain (SFD), so the spatial frequency dependency is obtained indirectly by means of the Hankel transform,^28^ which is only applicable in certain conditions, at the cost of losing all spatial information.

#### 2.3.1 Standard diffusion approximation

The first analytical model that we investigated is the standard diffusion approximation (SDA).^23^ The SDA is a model already used in many medical applications that works best in regimes in which scattering is predominant compared with absorption ($\mu_{s}^{\prime }\gg \mu_{a}^{\prime }$).^16^ For this reason, the SDA is more effective for light in the infrared spectrum because of the low absorption from biological tissue. The formula of light fluence (normalized to the incident power $P_{0}$) in the SFD for the diffuse approximation is given in the following equation:

$$\frac{\phi (z,f_{x})}{P_{0}}=A\cdot \exp(-\mu_{tr}\cdot z)+C\cdot \exp(-\mu_{\mathrm{eff}}^{\prime }(f_{x})\cdot z),$$ (4)

where A and C are constants derived by the choice of an appropriate boundary condition and R is a constant dependent on the effective reflection coefficient, which are given as

$$A=\frac{3\frac{\mu_{s}^{\prime }}{\mu_{tr}}}{\frac{\mu_{eff}^{\prime 2}}{\mu_{tr}^{2}}-1};\;C=\frac{-3\frac{\mu_{s}^{\prime }}{\mu_{tr}}(1+3R)}{\left(\frac{\mu_{\mathrm{eff}}^{\prime 2}(f_{x})}{\mu_{tr}^{2}}-1)(\frac{\mu_{\mathrm{eff}}^{\prime }(f_{x})}{\mu_{tr}}+3R\right)};\;R=\frac{1-R_{\mathrm{eff}}}{2(1+R_{\mathrm{eff}})}.$$ (5)

For the full derivation and explanation of the coefficients, we refer to Cuccia et al.^16^

#### 2.3.2 δ-P1 approximation

Because our SFDI instrumentation uses a light source that includes the visible spectrum and we are trying to detect photons that have short pathlengths, the SDA becomes less accurate as we are approaching the limits at which the model is still reliable. For this reason, we also considered a second analytical model. The δ-P1 approximation is a diffusion-based model that introduces a correction factor to account for photons that are non-diffuse. This correction factor allows for modeling fluence more accurately in those conditions in which the SDA becomes less reliable (e.g., for short distance photon propagation).^24^^,^^29^ The original δ-P1 model did not include dependency on the spatial frequency $f_{x}$,^29^ which was only introduced later, in the context of a doctoral thesis.^30^ The formula of light fluence (normalized to the incident power $P_{0}$) in the spatial frequency domain for the δ-P1 model is given in Eq. (6). For a complete derivation and explanation of the coefficients, we refer to the doctoral thesis of Seo.^30^

$$\frac{\phi_{AC}(z,f_{x})}{P_{0}}=\frac{C^{*}}{\mu_{\mathrm{eff}}^{\prime }-\mu_{tr}^{*}}[\exp(-\mu_{tr}^{*}z)-\exp(-\mu_{\mathrm{eff}}^{\prime }z)]+\frac{C^{*}}{\mu_{\mathrm{eff}}^{\prime }+\mu_{tr}^{*}}[\exp(-\mu_{tr}^{*}z)-\exp(-\mu_{\mathrm{eff}}^{\prime }(z+2z_{b}))],$$ (6)

where $\mu_{tr}^{*}=\mu_{a}+\mu_{s}^{*}=\mu_{a}+(1-g^{2})\mu_{s}$ as described in Carp et al.^29^ and the constants $C^{*}$ and $z_{b}$ are obtained by the application of the boundary condition, as described in the appendix of Seo’s doctoral thesis and are given as^30^

$$C^{*}=\frac{3\mu_{tr}\mu_{s}^{*}}{2\mu_{\mathrm{eff}}^{\prime }};\;z_{b}=\frac{2}{3\mu_{tr}}\frac{\left(1+R_{1}\right)}{\left(1-R_{1}\right)}.$$ (7)

#### 2.3.3 Modified δ-P1 approximation

In this study, we also derived our own spatial frequency-dependent δ-P1 equation based on the original δ-P1 equation^29^ and applied the same modification and boundary conditions made by Cuccia et al. in their derivation of the SDA in the spatial frequency domain.^16^ We call this derivation modified δ-P1, or mod-δ-P1. The equation of light fluence for the mod-δ-P1 approximation is shown in the following equation:

$$\frac{\phi (z,f_{x})}{P_{0}}=(1+A^{\prime })\exp(-\mu_{tr}^{*}\cdot z)+C^{\prime }\;\exp(-\mu_{\mathrm{eff}}^{\prime }z),$$ (8)

where the coefficients $A^{\prime }$ and $C^{\prime }$ are obtained by solving Boltzmann differential equation with the appropriate boundary conditions as

$$A^{\prime }=\frac{3\mu_{s}^{*}(\mu_{tr}^{*}+g^{*}\mu_{a})}{\left(\mu_{\mathrm{eff}}^{\prime 2}-\mu_{tr}^{*2}\right)};\;C^{\prime }=\frac{-A^{\prime }(1+\frac{2}{3}R^{\prime }\mu_{tr}\mu_{tr}^{*})+2R^{\prime }\mu_{tr}g^{*}\mu_{s}^{*}}{\left(1+\frac{2}{3}R^{\prime }\mu_{tr}\mu_{\mathrm{eff}}^{\prime }\right)};\;R^{\prime }=\frac{\left(1+R_{1}\right)}{\left(1-R_{1}\right)}.$$ (9)

For a full derivation and explanation of the coefficients, we refer to Appendix A.

**Fig. 2:**
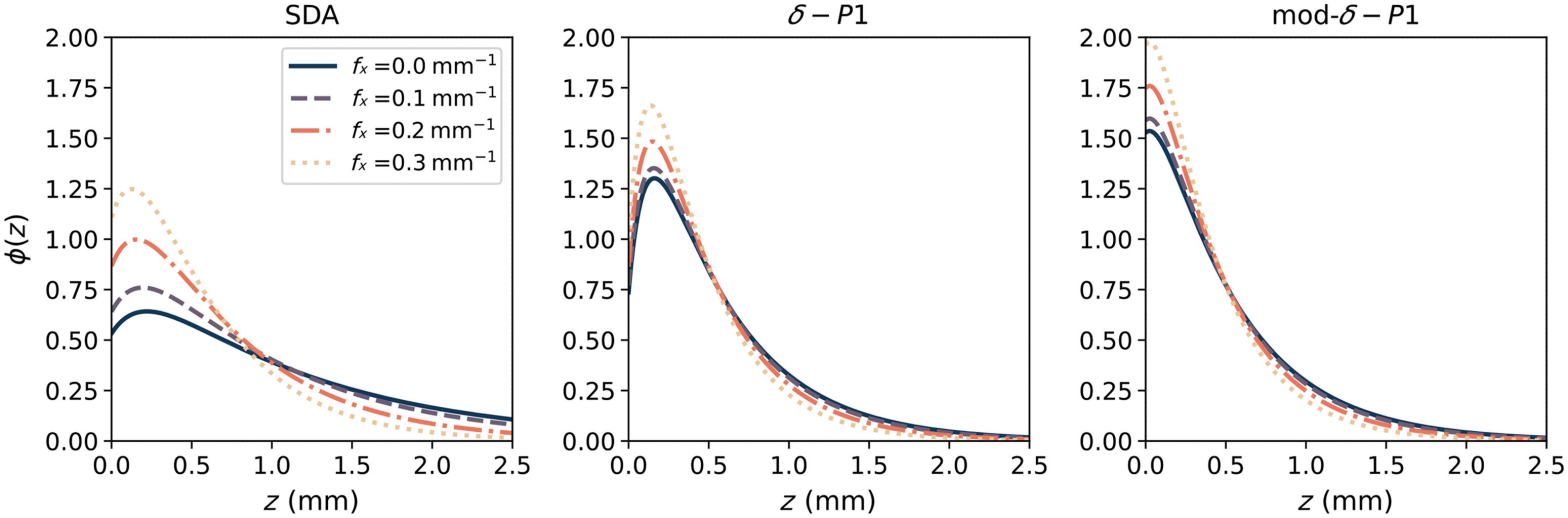
Comparison between fluence (ϕ) models using SDA (left), the original δ-P1 approximation (center), and our mod-δ-P1 derivation (right). The values of ϕ were normalized to their total integral over z. In each plot, the effect of the spatial frequency ($f_{x}$) on the fluence distribution is also shown. All models of ϕ in the figure were simulated on an homogeneous geometry with the same optical properties: $\mu_{a}=0.05\;{\mathrm{mm}}^{-1}$ and $\mu_{s}^{\prime }=5\;{\mathrm{mm}}^{-1}$.

A comparison between the SDA and the two δ-P1 models of fluence are given in Fig. 2, which shows the values of ϕ(z), normalized to the total integral of ϕ over z. The models were calculated for optical properties $\mu_{a}=0.05\;{\mathrm{mm}}^{-1}$ and $\mu_{s}^{\prime }=5\;{\mathrm{mm}}^{-1}$. Figure 2 also shows how the spatial frequency changes the distribution of the photons, with higher values of $f_{x}$ having photons more concentrated near the surface.

## 3 Materials and Methods

### 3.1 Thin Phantoms Fabrication

To test the validity of the models, we manufactured silicone multi-layered optical phantoms with controlled scattering properties and thicknesses. These multi-layer phantoms were used to acquire experimental data to which the models were compared. India ink was used as an absorber because of its relatively flat absorption spectrum across the entire measured range. Because this study is focused on developing a model of light scattering, we want to minimize the spectral features generated by absorbers, which could alter the scattering spectrum in the case of cross-talk during the processing of the data. The ink was mixed in a sufficiently large volume of unpolymerized silicone with a concentration of 20  mg/100  ml, before splitting it into different batches of phantoms, so that the absorption would remain consistent across all manufactured phantoms. Given the ink concentration used, we expected a flat absorption value of about $0.0167\;{\mathrm{mm}}^{-1}$,^31^^,^^32^ which was also independently measured using optical methods (3.2.1), to account for imprecisions in the fabrication process and the reliability of the used phantom recipe. The silicone was divided in two to fabricate two different batches of phantoms with different scattering particles. The scatterers were mixed in the silicone curing agent and sonicated to break down any aggregation of particles that would change their size distribution. Then the curing agent was mixed with the unpolymerized silicone in a ratio of 1:5, and a vacuum chamber was used to remove any air bubbles formed in the process.^31^ From each batch, five thin phantoms, with a thickness approximately in the range 0.1 to 1 mm, were obtained using a syringe to measure small volumes of liquid silicone (0.5 to 3 ml), poured onto a petri dish of 5 cm in diameter, and left to spread by resting on a level surface. The thickness of each phantom was then measured using a micrometer, taking multiple readings in different spots (N=10). A summary of the value of thickness (mean and standard deviation) is given in Table 1. The rest of the silicone was poured into a container to obtain a thick homogeneous phantom of about 20 to 30 mm in thickness. The scattering agent used in the first batch was aluminum oxide (${\mathrm{Al}}_{2}O_{3}$) to emulate the underlying wound morphology, with an average particle size of 5  μm, in a concentration of 3  g/100  ml. The scattering coefficient measured at 650 nm is expected to be $∼0.8\;{\mathrm{mm}}^{-1}$, with a scattering slope of ∼0.15.^33^ The scattering agent used in the second batch was titanium oxide (${\mathrm{TiO}}_{2}$) to emulate cellular proliferation, with an average particle size of 200 nm, in a concentration of 130  mg/100  ml. The scattering coefficient measured at 650 nm is expected to be $∼1.78\;{\mathrm{mm}}^{-1}$,^31^ with a scattering slope of ∼1.2.^33^

**Table 1:** Thickness of the thin ${\mathrm{TiO}}_{2}$ phantoms, measured with a micrometer in 10 different spots to calculate the average and standard deviation.

Phantom 1	Phantom 2	Phantom 3	Phantom 4	Phantom 5
0.130 ± 0.004 mm	0.269 ± 0.014 mm	0.490 ± 0.022 mm	0.675 ± 0.040 mm	1.149 ± 0.065 mm

### 3.2 Data Acquisition

#### 3.2.1 Phantoms characterization

First, the optical properties of the two batches were measured on the 30 mm thick homogeneous phantoms using a handheld SFDI imager, capable of getting spatial information at five spectral bands in the visible range (450 to 630 nm).^34^ Because there is no spatial heterogeneity in the phantoms’ optical properties, a rectangle was drawn on the center of the SFDI images, and the spatial average was calculated. The same measurements were also independently repeated for validation using spatial frequency domain spectroscopy (SFDS), which is a probe-based system capable of doing measurements with high spectral resolution in the range 400 to 1000 nm on a single point in space.^35^ Both SFDI and SFDS measurements were calibrated with respect to the same reference homogeneous phantom of known optical properties. The SFDS measurements were taken in three different spots for each phantom, but no significant changes were detected, which shows the homogeneous composition of the targets. The scattering coefficients at 650 nm are $∼2.07\;{\mathrm{mm}}^{-1}$ for ${\mathrm{TiO}}_{2}$ and $0.8\;{\mathrm{mm}}^{-1}$ for ${\mathrm{Al}}_{2}O_{3}$, and the absorption coefficients of both were within 0.016 to $0.026\;{\mathrm{mm}}^{-1}$. Eventual differences from the values expected from the phantom recipes in Sec. 3.1 are to be attributed to imprecisions in the fabrication process. The $\mu_{a}$ and $\mu_{s}^{\prime }$ of the two batches are reported in Table 2 at three spectral bands (458, 536, and 626 nm), with the respective ratio between the $\mu_{s}^{\prime }$ of the top and bottom layers (scattering contrast).

**Table 2:** Optical properties of the two batches measured on 30 mm-thick phantoms, reported at three spectral bands. The first batch contains titanium oxide particles for scattering (row 1-2), and the second one contains aluminum oxide (row 3-4). Both batches contain India Ink for absorption. The ratio of the $\mu_{s}^{\prime }$ of the top layer to the $\mu_{s}^{\prime }$ of the bottom layer is reported on the bottom row.

		458 nm	536 nm	626 nm
${\mathrm{TiO}}_{2}$	$\mu_{a}$ (${\mathrm{mm}}^{-1}$)	0.0322	0.0370	0.0427
(Top layer)	$\mu_{s}^{\prime }$ (${\mathrm{mm}}^{-1}$)	3.4319	2.9269	2.3436
${\mathrm{Al}}_{2}O_{3}$	$\mu_{a}$ (${\mathrm{mm}}^{-1}$)	0.0215	0.0226	0.0242
(Bottom layer)	$\mu_{s}^{\prime }$ (${\mathrm{mm}}^{-1}$)	0.8675	0.8322	0.7830
	$\mu_{s_{\text{top}}}^{\prime }/\mu_{s_{\mathrm{bot}}}^{\prime }$	3.9562	3.5170	2.9930

#### 3.2.2 Multi-frequencies measurements

After having characterized the two batches, data were acquired on multi-layered phantoms using the SFDI imager previously described. The two-layered phantoms were obtained by placing each of the thin phantoms over the thick homogeneous phantom from the opposite batch to have two layers with distinct scattering properties. To improve adhesion between the layers and to reduce the refraction index mismatch due to the presence of air bubbles, ultrasound gel was spread between the phantoms and was squeezed out as much as possible. The ${\mathrm{Al}}_{2}O_{3}$ thick phantom was used as the bottom layer, and the ${\mathrm{TiO}}_{2}$ thin phantoms were the top layer. A total of 11 spatial frequencies were acquired for each dataset, from $0\;{\mathrm{mm}}^{-1}$ (planar illumination) to $0.5\;{\mathrm{mm}}^{-1}$, in incremental steps of $0.05\;{\mathrm{mm}}^{-1}$. The two-layered phantom measurements were calibrated to the same reference used in Sec. 3.2.1 on homogeneous phantoms, minimizing the introduction of calibration errors.

### 3.3 Data Processing

**Fig. 3:**
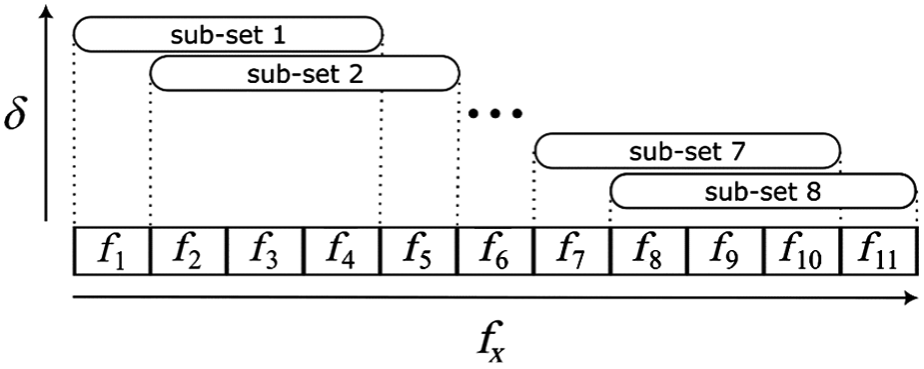
Subdivision of the dataset containing 11 spatial frequencies ($f_{x}$) in 8 smaller, partially overlapped sub-sets, containing $4f_{x}$ each. The sub-sets have different penetration depths (δ), which are estimated considering the average $f_{x}$ of the sub-sets ($\left\langle f_{x}\right\rangle$), with lower $\left\langle f_{x}\right\rangle$ resulting in higher δ.

Our new multi-frequencies processing approach consists of subdividing the dataset into several smaller, partially overlapping sub-sets containing four frequencies each, with increasing values of $f_{x}$, as seen in Fig. 3. Each sub-set is individually processed to obtain $\mu_{a}$ and $\mu_{s}^{\prime }$ values, which have different penetration depths, as defined in Sec. 2. The average $f_{x}$ of each subset is used in the fluence calculation, denoted as $\left\langle f_{x}\right\rangle$ in the text. The SFDI data were processed assuming a homogeneous volume using the procedure described in Cuccia et al.:^16^ the amplitude of the modulated patterns was extracted and a homogeneous reference phantom with known optical properties was used for calibration to obtain the measurements of diffuse reflectance ($R_{d}$). A white Monte Carlo model of $R_{d}$ is then used in an iterative algorithm that changes the input parameters $\left(\mu_{a},\mu_{s}^{\prime }\right)$ according to an optimization strategy, until the $R_{d}$ of the model matches the $R_{d}$ measured on the target, giving as a result the $\left(\mu_{a},\mu_{s}^{\prime }\right)$ pair of the measured target.

## 4 Results

### 4.1 Measured $\mu_{s}^{\prime }$

**Fig. 4:**
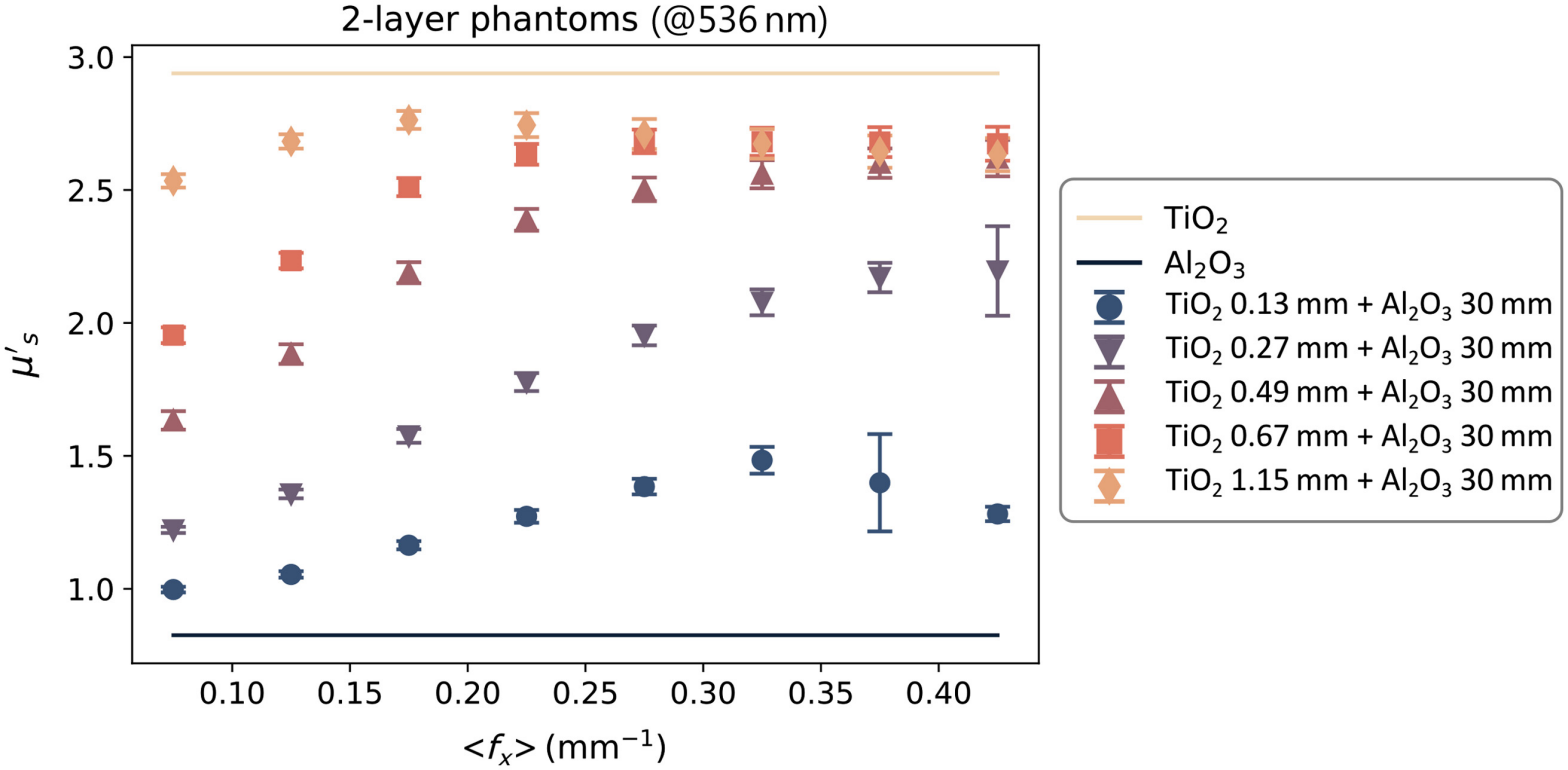
Scattering coefficients ($\mu_{s}^{\prime }$) of the five two-layered phantoms (colored markers), measured at 536 nm. The solid lines are the $\mu_{s}^{\prime }$ of the top layer (light orange) and bottom layer (dark blue). The data on the horizontal axis represent the average spatial frequency $\left\langle f_{x}\right\rangle$ of the eight sub-sets into which the data were divided.

A sample of the data measured in Sec. 3.2.2 and processed assuming homogeneous tissue, as described in Sec. 3.3, is shown in Fig. 4. The figure contains data from a single spectral band (536 nm) because the measurements at each wavelength are independent from one another. The graph shows the scattering coefficients of the five two-layered phantoms and how they change with $\left\langle f_{x}\right\rangle$. The horizontal axis is the average of the spatial frequencies contained in each of the eight sub-sets. The $\mu_{s}^{\prime }$ of the top layer (${\mathrm{TiO}}_{2}$ – light orange line) and bottom layer (${\mathrm{Al}}_{2}O_{3}$ – dark blue line) are included for comparison. The $\mu_{s}^{\prime }$ of these two layers are assumed to be constant across the spatial frequencies and were obtained by measuring it on the thick homogeneous phantoms using the same SFDI system.

### 4.2 Fluence-Based Models

**Fig. 5:**
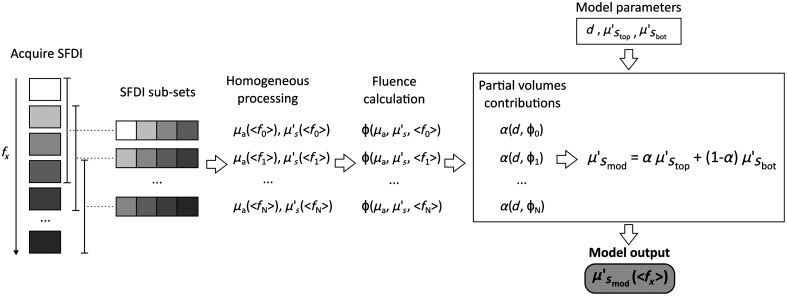
Summary of the procedure to acquire depth-sensitive data using multi-frequency SFDI and use it as input to the two-layer scattering model. SFDI images are acquired at several spatial frequencies and divided in sub-sets containing four frequencies each. From each sub-set, optical properties are estimated and used to calculate the homogeneous fluence rate ϕ. Values of ϕ are then used in combination with the model parameters to estimate the partial volume contributions α and obtain a model of $\mu_{s}^{\prime }(\langle f_{x}\rangle )$.

**Fig. 6:**
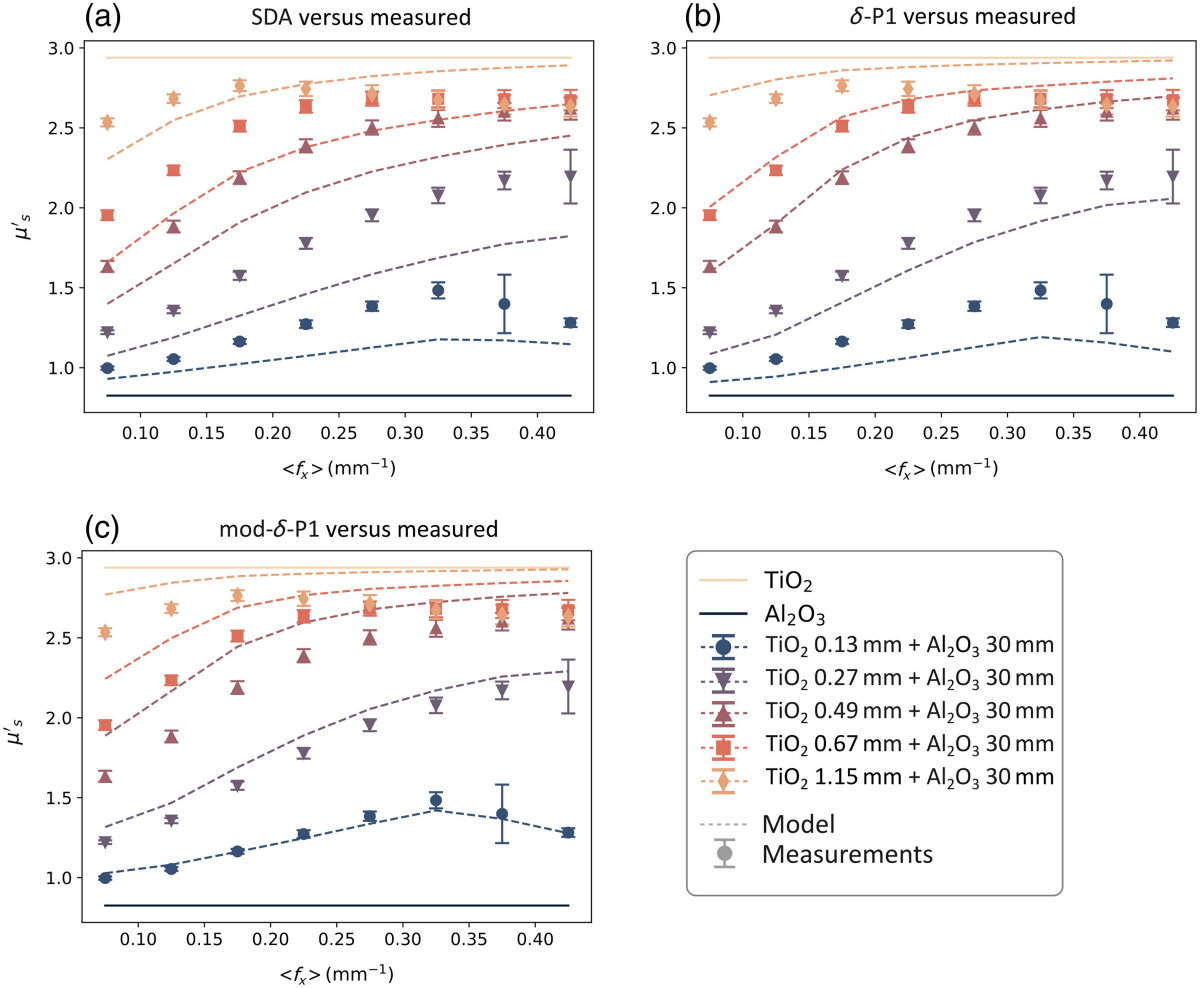
Comparison of the three fluence-based two-layer models (dashed lines) with the data (markers) measured at 536 nm. The top (light orange) and bottom (dark blue) solid lines are the scattering coefficients of the ${\mathrm{TiO}}_{2}$ and ${\mathrm{Al}}_{2}O_{3}$ layers, respectively. The three models are (a) SDA, (b) the original δ-P1 approximation, and (c) our mod-δ-P1 approximation.

Using the values of $\mu_{s_{\text{top}}}^{\prime }$ and $\mu_{s_{\mathrm{bot}}}^{\prime }$ measured in Sec. 3.2.1 and the phantom thickness (d) reported in Table 1, the expected values of $\mu_{s}^{\prime }(f_{x})$ of the two-layered phantoms were modelled using Eqs. (1) and (3). The $\mu_{a}$ and $\mu_{s}^{\prime }$ measured on the layered phantoms, as described in Sec. 3.2.2, were used to calculate the fluence ϕ used in Eq. (3) with the three models presented in Sec. 2.3. The procedure is summarized in the flowchart in Fig. 5. In Fig. 6, a comparison between the modeled $\mu_{s}^{\prime }$ values and the experimental measurement on phantoms at 536 nm is shown for both SDA and the two δ-P1 models.

### 4.3 Models’ Performance

**Fig. 7:**
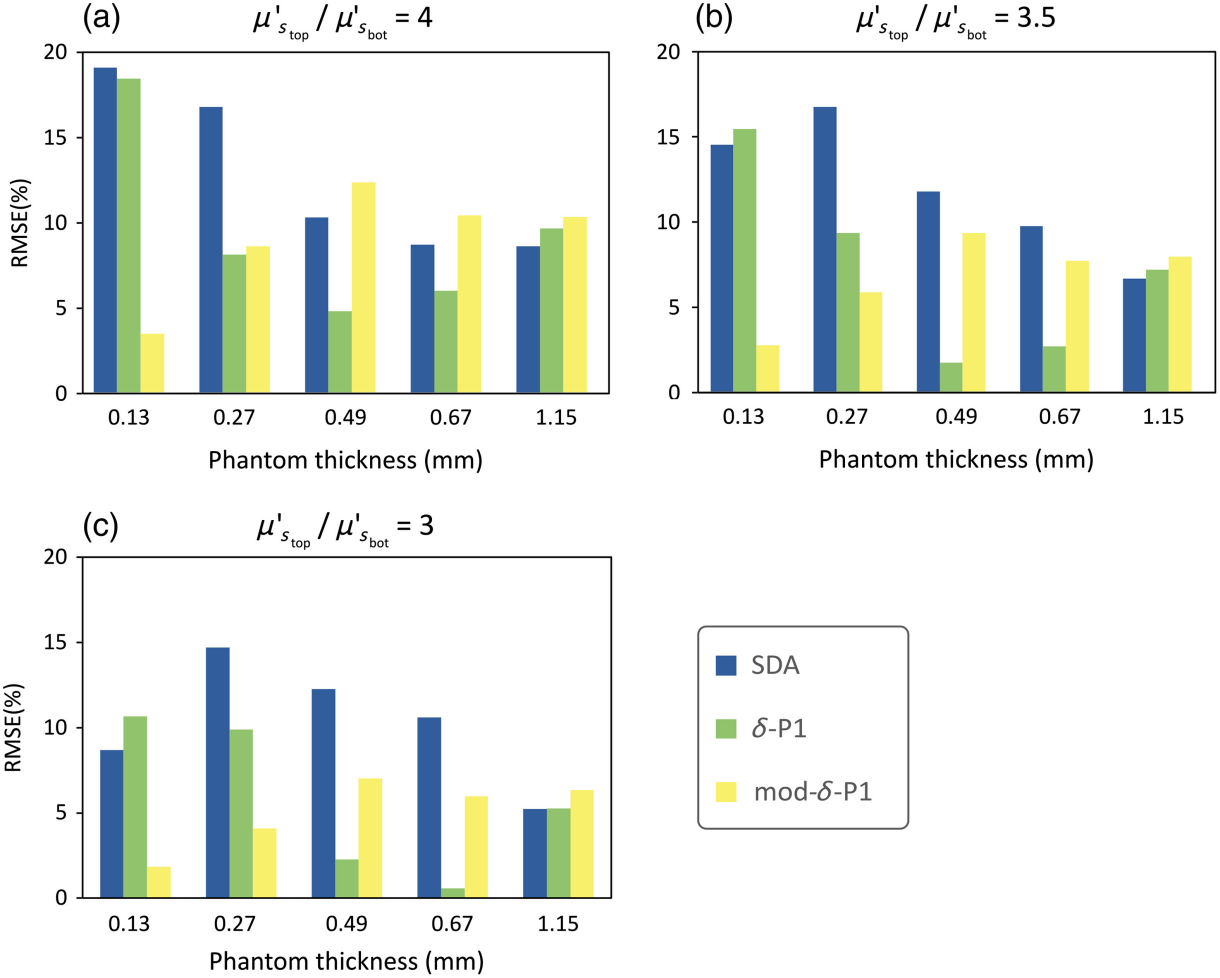
RMSPE calculated on the difference between the scattering coefficient of the three fluence-based two-layer models and the experimental measurements. Each figure has a different scattering contrast, defined as the ratio $\mu_{s_{\text{top}}}^{\prime }/\mu_{s_{\mathrm{bot}}}^{\prime }$ and shows the RMSPE of the three models for the five top layer thicknesses (in mm). The scattering ratios are approximately a=4, b=3.5, and c=3.

To obtain an objective evaluation of the accuracy of the two-layered models of $\mu_{s}^{\prime }$ and compare their performance, the RMSPE was calculated with respect to the measurements described in Sec. 4.1 for each of the models and each of the phantom thicknesses using the following equation:

$$\mathrm{RMSPE}=\sqrt{\frac{1}{N}\overset{N}{\underset{i=1}{\sum }}{\left(\frac{\mu_{s_{\mathrm{meas}}}^{\prime }(\langle f_{i}\rangle )-\mu_{s_{\mathrm{mod}}}^{\prime }(\langle f_{i}\rangle )}{\mu_{s_{\mathrm{meas}}}^{\prime }(\langle f_{i}\rangle )}\right)}^{2}}*100,$$ (10)

where N is the number of average spatial frequencies contained in the dataset (N=8), $\mu_{s_{\mathrm{meas}}}^{\prime }$ is the measured scattering coefficient, and $\mu_{s_{\mathrm{mod}}}^{\prime }$ is the scattering coefficient modeled using Eq. (1). The chart in Fig. 7 summarizes the RMSPE of three datasets having different scattering contrasts, defined as the ratio $\mu_{s_{\text{top}}}^{\prime }/\mu_{s_{\mathrm{bot}}}^{\prime }$.

## 5 Discussion

By observing the $\mu_{s_{\mathrm{meas}}}^{\prime }$ data in Fig. 4, we can make a few observations. First, the value of the $\mu_{s}^{\prime }$ coefficients measured on the two-layered phantoms are contained between $\mu_{s_{\text{top}}}^{\prime }$ and $\mu_{s_{\mathrm{bot}}}^{\prime }$, which is consistent with the hypothesis made in Sec. 2 of $\mu_{s_{\mathrm{mod}}}^{\prime }$ being a combination of the other two scattering coefficients. Second, with an increasing thickness of the thin layer, the $\mu_{s_{\mathrm{meas}}}^{\prime }$ of the two-layered phantoms becomes closer to $\mu_{s_{\text{top}}}^{\prime }$. This is to be expected because the relative contribution of the top layer to the overall scattering becomes greater the thicker the layer is. Similarly, with the increase of the average $f_{x}$, the $\mu_{s_{\mathrm{meas}}}^{\prime }$ of the two-layered phantoms becomes closer to $\mu_{s_{\text{top}}}^{\prime }$. This behavior is also in line with the SFDI property shown in Sec. 2.2, where higher spatial frequencies have less penetration depth, so the relative contribution from the top layer becomes higher. A last consideration that can be done is that the dependency of the two-layered $\mu_{s}^{\prime }$ over $\left\langle f_{x}\right\rangle$ seems to be non-linear in nature and tends to “saturate” for high enough values of $\left\langle f_{x}\right\rangle$ or layer thickness. This saturation of $\mu_{s}^{\prime }$ can be explained by assuming that the penetration depth of light (δ) becomes smaller than the thickness of the top layer, so we are effectively measuring a single layer instead of two. Looking at the two-layer scattering models in Fig. 6, it is possible to determine where the underlying fluence models are the most accurate and what their limitations are. The SDA model [Fig. 6(a)] seems to be more accurate for larger thicknesses, but it tends to underestimate the contribution of the top layer, especially at low $\left\langle f_{x}\right\rangle$. This is expected, as SDA assumes that the photons have long enough pathlengths to be considered diffuse (distance from the light source $\gg 1/\mu_{t}$). This behaviour is also evident from the fluence plots in Fig. 2 that show a larger concentration of photons deeper in the tissue compared to the other models. The original δ-P1 model [Fig. 6(b)] seems to have an accuracy similar to the SDA in the extreme cases (d<0.1  mm, d>1  mm), but it is more accurate in the middle range (0.3  mm<d<0.7  mm) and does not overestimate the contribution of the bottom layer at low $f_{x}$, thanks to the additional correction term that models photons with a low number of scattering events/short pathlengths. The modified δ-P1 model [Fig. 6(c)] is the most accurate for very thin layers (d<0.2  mm), but it seems to over-estimate the contribution of the top layer and becomes less accurate for thickness d>0.5  mm. In Fig. 7, we compare the performance of the models by looking at the RMSPE calculated for all thicknesses and three different spectral bands. Because the two scattering agents (${\mathrm{TiO}}_{2}$ and ${\mathrm{Al}}_{2}O_{3}$) have a large difference in the scattering slope, it is possible to choose bands far enough apart, so the ratio $\mu_{s_{\text{top}}}^{\prime }/\mu_{s_{\mathrm{bot}}}^{\prime }$ is different and we can do an evaluation with different contrasts between the top and bottom layer. In the three bands that we reported (458, 536, and 626 nm), the ratio is equal to approximately 4, 3.5, and 3, respectively.

A first consideration is that all three models have a similar performance for d>1  mm, which might be an additional indication that we are exceeding the limits of the two-layers model and measuring only the top layer. Second, both δ-P1 models perform better than the SDA in most cases, with the original δ-P1 model performing better in the range 0.3  mm<d<0.7  mm and our modified δ-P1 model performing better in the range d<0.3  mm. Third, the RMSPE seems to improve in all models for a smaller $\mu_{s_{\text{top}}}^{\prime }/\mu_{s_{\mathrm{bot}}}^{\prime }$ ratio (i.e., less contrast between the layers), which is the opposite of what was initially expected. However, when considering that this two-layer approach is based on measurements that assume a homogeneous fluence distribution, it becomes unsurprising that errors in estimating the layer-specific scattering coefficients would increase the more we deviate from this assumption (i.e., for increasing difference in optical properties).

Nevertheless, these preliminary results remain extremely encouraging in the context of the target application (assessment of wound healing), in which we are interested in detecting a difference in $\mu_{s}^{\prime }$ in very thin layers of cells (0.1 to 0.2 mm). In particular, our modified δ-P1 model has an overall good performance (RMSPE<10%), which becomes even better in the physiological range of interest (RMSPE<5%). The objective of this initial investigation was to evaluate how closely homogenous fluence models could match SFDI measurement of layered media over differing penetration depths.

In Sec. 2.3, we stated our motivations behind the selection of the analytical models of fluence analyzed in this study (i.e., computation speed and ease of implementation). However, any model of light transport suitable for SFDI, which includes spatial frequency information, could theoretically be used without affecting the validity of the two-layer model.^25^^–^^27^ This allows for future improvement by adopting a model that is capable of representing the fluence of light more accurately in different conditions and for different geometries and optical properties.

Multilayer fluence models in the spatial frequency domain do already exist^36^ and can be utilized as a second stage optimization, using the results from the homogeneous models as initial estimates of layer thickness and layer specific optical properties.

The current work was aimed at modeling the depth-resolved multi-frequencies SFDI data in the most accurate way possible. However, the method still relies on pre-existent knowledge about the optical and geometrical parameters, which are the ones containing useful information for diagnostic purposes. For the method to be of practical use, an iterative inverse-solving algorithm will be implemented. The algorithm will allow for estimating the scattering parameters and layer thickness from the raw multi-frequencies SFDI measurements, providing the information most useful to aid clinicians in diagnosis. Furthermore, by combining the frequency-dependent depth estimation used in this work with the wavelength-dependent depth estimation from a previous work,^20^ we will be able to offer a multi-purpose suite of optical tools for the analysis of various skin conditions, ranging from burn wounds to melanoma and everything else in-between.

## 6 Conclusions

We have presented an approach to processing SFDI data using different sub-sets of spatial frequencies to obtain datasets that have different depth information. We then made use of this depth-enhanced data to develop and validate a two-layer model of scattering for thin layers, aimed at mimicking the physiology of a healing wound. The model is based on the relative layer contribution to $\mu_{s}^{\prime }$, calculated through an integral function of the light fluence. The performance of three analytical models of fluence was analyzed in the study, but the method itself is model agnostic and can be used with any model of fluence, leaving room for further improvement. The performance of the analytical model themselves looks promising, with the δ-P1 models overall working better than the SDA model. The proposed mod-δ-P1 model also has an excellent performance for very thin layers, which is especially interesting for the target application of this study, which is to measure layers of skin that are on the order of 0.1 to 0.2 mm.

## 7 Appendix A

The initial derivation follows the same procedure seen in the original δ-P1 model.^29^ By inserting the δ-P1 phase function in the Boltzmann transport equation, we obtain the following governing equations:

∇2ϕd(r)−μeff2ϕd(r)=−3μs*μtrq(r,z)+3g*μs*∇q(r,z)·z^, (11)

j(r)=−13μtr[∇ϕd(r)−3g*μs*q(r,z)z^], (12)

where $\mu_{tr}=\mu_{a}+\mu_{s}^{\prime }$, $\mu_{s}^{*}=\mu_{s}(1-g^{2})$, $g^{*}=g/(g+1)$, and $\mu_{\mathrm{eff}}=\sqrt{3\mu_{a}\mu_{tr}}$. The source term that we introduce is of the kind

q(r,z)=q(r)(1−Rs)exp(−μt*·z)δ(1−ω^z^), (13)

where $R_{s}$ is the specular reflectance and $q_{0}(r)$ is the irradiance of the light source. For planar illumination, we would have a constant value $q_{0}(r)=q_{0}$. This is where our approach differs from the original δ-P1 derivation, as we introduce a sinusoidally modulated light source, as seen in Cuccia et al.,^16^ which is given as

$$q(z)=q_{0}(z)\cos(k_{x}x+A)\;\cos(k_{y}y+B).$$ (14)

We can then make the same considerations about the linearity of the medium, giving in response a sinusoidal fluence rate with no phase shift, given as

$$\phi (z)=\phi_{0}(z)\cos(k_{x}x+A)\cos(k_{y}y+B).$$ (15)

By introducing Eqs. (14) and (15) in Eq. (11), after the opportune simplifications, we obtain a 1-D differential equation in the depth dimension, given as

$$\frac{d^{2}\varphi_{0}(z)}{dz^{2}}-\mu_{\mathrm{eff}}^{\prime 2}\varphi_{0}(z)=-3\mu_{s}^{*}\mu_{t}q_{0}(z)+3g^{*}\mu_{s}^{*}\frac{dq_{0}(z)}{dz},$$ (16)

where $\mu_{\mathrm{eff}}^{\prime 2}=(\mu_{\mathrm{eff}}^{2}+k_{x}^{2}+k_{y}^{2})$ is the modified effective scattering coefficient, which is the term that introduces the spatial frequency dependence in the fluence equation, as seen in the standard diffuse approximation.^16^

From here on, we can continue following the derivations steps found in the appendix of Carp et al.,^29^ solving the differential equation in a manner similar to the case of planar illumination on a semi-infinite geometry, but substituting the coefficient $\mu_{\mathrm{eff}}^{2}$ with $\mu_{\mathrm{eff}}^{\prime 2}$. There are two boundary conditions required. First, because of the conservation of energy, the intensity of the diffuse light field must be zero in regions at a large distance from the source, given as

$$\phi_{d}(r){|}_{r\to \infty }\to 0.$$ (17)

The second boundary condition is given by the conservation of the diffuse flux component normal to the surface, which is given as

[ϕd(r,z)−23μtrR′∇ϕd(r,z)z^]z=0=−2μtrR′g*μs*  q(r,z)|z=0, (18)

where $R^{\prime }=\frac{\left(1+R_{1}\right)}{\left(1-R_{1}\right)}$, where $R_{1}$ is the first moment of the Fresnel reflection coefficient in non-polarized light. This implementation of $R^{\prime }$ is just an approximation of the correct term $\frac{\left(1+R_{2}\right)}{\left(1-R_{1}\right)}$ that makes use of the first two moments of the Fresnel coefficient. We chose to use this implementation because it gives a better approximation in proximity of the source, at the cost of reducing the model accuracy in the far field. Usually, the $R^{\prime }$ coefficient is obtained directly from a polynomial expression, as seen in Carp et al.^29^ For the one-dimensional case, the equation of the second boundary condition is reduced to

$${\left[\phi_{d}(z)-\frac{2}{3\mu_{tr}}R^{\prime }\frac{d\phi_{d}(z)}{dz}\right]}_{z=0}=-\frac{2}{\mu_{tr}}R^{\prime }g^{*}\mu_{s}^{*}q_{0}(1-R_{s}).$$ (19)

The boundary conditions allow for solving Eq. (16) and finding the diffuse fluence rate, normalized to the incident power $P_{0}(1-R_{s})$, with $R_{s}$ being the specular reflectance, which is given as

$$\frac{\phi_{d}(z,f_{x})}{P_{0}(1-R_{s})}=A^{\prime }\;\exp(-\mu_{tr}^{*}\cdot z)+C^{\prime \;\exp(-\mu_{\mathrm{eff}}^{\prime }z)},$$ (20)

where

$$A^{\prime }=\frac{3\mu_{s}^{*}(\mu_{tr}^{*}+g^{*}\mu_{a})}{\left(\mu_{\mathrm{eff}}^{\prime 2}-\mu_{tr}^{*2}\right)};\;C^{\prime }=\frac{-A^{\prime }(1+\frac{2}{3}R^{\prime }\mu_{tr}\mu_{tr}^{*})+2R^{\prime }\mu_{tr}g^{*}\mu_{s}^{*}}{\left(1+\frac{2}{3}R^{\prime }\mu_{tr}\mu_{\mathrm{eff}}^{\prime }\right)}.$$ (21)

Finally, by adding the collimated (non-diffuse) fluence contribution to Eq. (20), which is given as

$$\frac{\phi_{c}(z,f_{x})}{P_{0}(1-R_{s})}=\exp(-\mu_{tr}^{*}\cdot z),$$ (22)

we obtain

$$\frac{\phi (z,f_{x})}{P_{0}(1-R_{s})}=\frac{\phi_{c}+\phi_{d}}{P_{0}(1-R_{s})}=(1+A^{\prime })\exp(-\mu_{tr}^{*}\cdot z)+C^{\prime \;\exp(-\mu_{\mathrm{eff}}^{\prime }z)},$$ (23)

which is in agreement with the solution presented in Eqs. (8) and (9).

## 8 Appendix B: Additional Figures

**Fig. 8:**
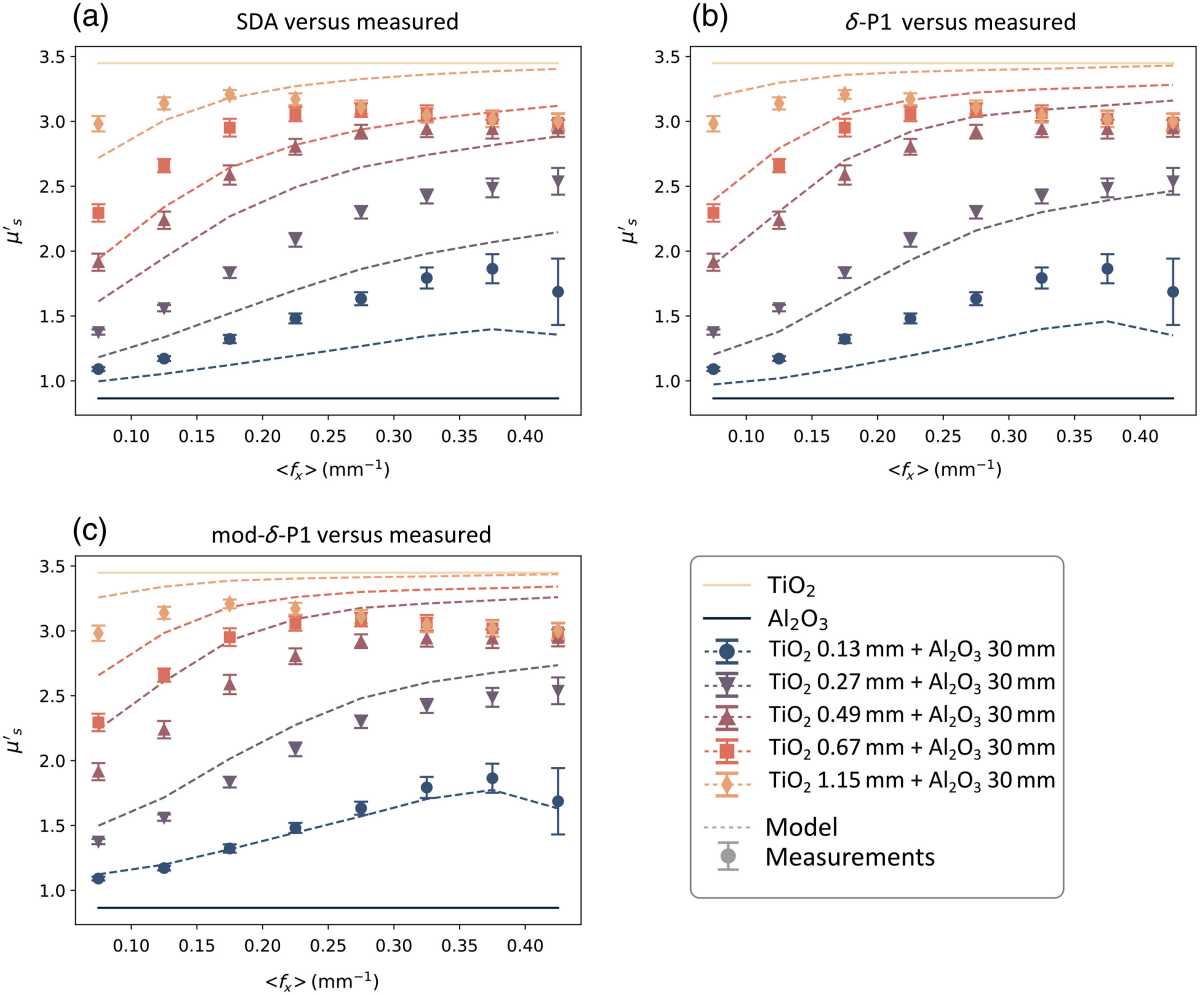
Comparison of the three fluence-based two-layer models (dashed lines) with the data (markers) measured at 458 nm. (a) SDA, (b) δ-P1 approximation, and (c) mod-δ-P1 approximation.

**Fig. 9:**
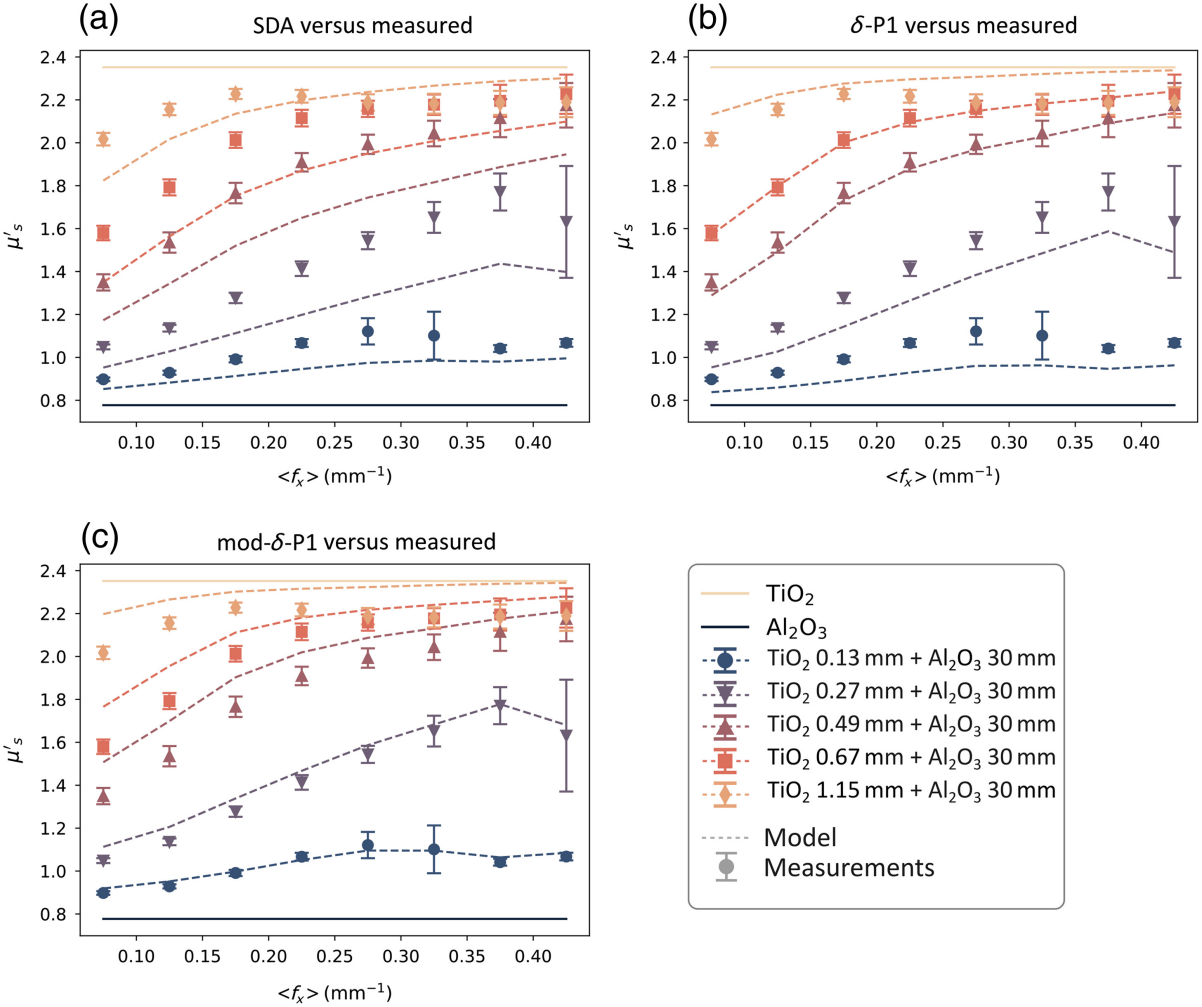
Comparison of the three fluence-based two-layer models (dashed lines) with the data (markers) measured at 626 nm. (a) SDA, (b) δ-P1 approximation, and (c) mod-δ-P1 approximation.

**Fig. 10:**
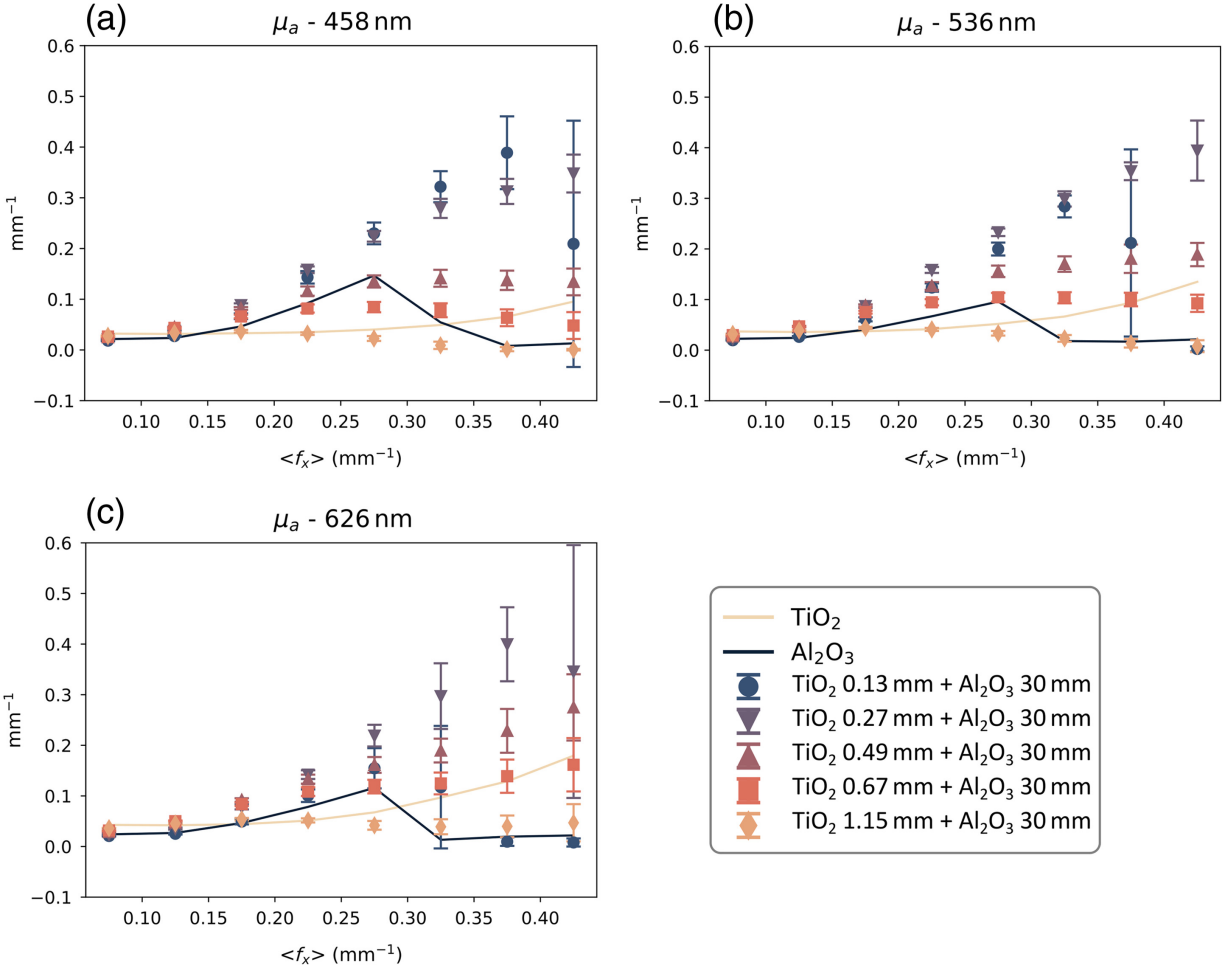
Absorption coefficient ($\mu_{a}$) measured on homogeneous phantoms (solid lines) and two-layered phantoms (markers) at three wavelengths, with the respective scattering contrast of the two-layered phantoms $\mu_{s_{\text{top}}}^{\prime }/\mu_{s_{\mathrm{bot}}}^{\prime }$ reported in parenthesis: (a) 458 nm (4), (b) 536 nm (3.5), and (c) 626 nm (3).

Additional data from the study is reported in this appendix and is shown in Figs. 8–10. In the main text of the paper, the results from the two-layer phantom combinations were presented at 536 nm as a demonstration of the three different models’ performance over increasing spatial frequency sets (Fig. 6). Here, Figs. 8 and 9 also show the same model performance relative to the measured bulk scattering results at 458 and 626 nm, which have a scattering contrast ratio between the layers of approximately 4 and 3, respectively.

A particular consideration could be given to the absorption coefficient measurements shown in Fig. 10. Given the manufacturing procedure of the phantoms, we expect a similar absorption in both the ${\mathrm{TiO}}_{2}$ and ${\mathrm{Al}}_{2}O_{3}$ phantoms, which is reflected in the measurements at low $\left\langle f_{x}\right\rangle$. However, with increasing $\left\langle f_{x}\right\rangle$, we begin to see an increase in $\mu_{a}$ in the multi-layered phantoms, especially the ones with the smallest thickness. We believe that this unexpected deviation is due to a combination of (1) the low sensitivity of absorption at high spatial frequencies (as previously shown in a study from Cuccia^16^) and (2) the low signal to noise ratio (SNR) of the spatial frequency dependent reflectance at high spatial frequencies, as can be seen by the large variance of the measurements. This unsettling propagation of error in $\mu_{a}(\langle f_{x}\rangle )$ estimation may be a source of concern regarding the reliability of the proposed models and methods. We found that absorption contributes minimally, if not negligibly, to the fluence estimation at higher spatial frequencies and, subsequently, to this fluence-based approach to interpreting the partial volume contribution of layer-specific scattering. In this higher spatial frequency range, both $\left\langle f_{x}\right\rangle$ and scattering become the dominant factors. Nevertheless, further investigation is warranted to determine the exact source of this unexpected variation in $\mu_{a}(\langle f_{x}\rangle )$ as it could benefit future instrumentation design (i.e., impact of a better SNR, dynamic range, and calibration methods in measurements) and model development (i.e., more robust light transport models to reflect increasingly sub-diffusive absorption events).

## Data Availability

All relevant code, data, and materials are available from the authors upon reasonable request. Correspondence and requests should be addressed to the corresponding author.

## References

[1] Bohjanen K., “Structure and functions of the skin,” in Clinical Dermatology, Soutor C. Hordinsky M. K., Eds., McGraw-Hill Education, New York, New York (2017).

[2] Velnar T. Bailey T. Smrkolj V., “The wound healing process: an overview of the cellular and molecular mechanisms,” J. Int. Med. Res. 37(5), 1528–1542 (2009). JGIMEJ 0884-8734 10.1177/147323000903700531 19930861

[3] Schultz G. S. et al., “Wound bed preparation: a systematic approach to wound management,” Wound Repair Regen. 11 Suppl 1, S1–28 (2003). 10.1046/j.1524-475x.11.s2.1.x 12654015

[r4] Falanga V., “Classifications for wound bed preparation and stimulation of chronic wounds,” Wound Repair Regen. 8(5), 347–352 (2000). 10.1111/j.1524-475X.2000.00347.x 11115147

[5] Grey J. E. Enoch S. Harding K. G., “ABC of wound healing wound assessment,” BMJ 332(7536), 285–288 (2006). 10.1136/bmj.332.7536.285 16455730 PMC1360405

[6] Bloemen M. C. T. Van Zuijlen P. P. M. Middelkoop E., “Reliability of subjective wound assessment,” Burns 37(4), 566–571 (2011). BURND8 0305-4179 10.1016/j.burns.2011.02.004 21388743

[7] Yafi A. et al., “Postoperative quantitative assessment of reconstructive tissue status in a cutaneous flap model using spatial frequency domain imaging,” Plast. Reconstr. Surg. 127(1), 117–130 (2011). 10.1097/PRS.0b013e3181f959cc 21200206 PMC3017473

[r8] Crane N. J. et al., “Evidence of a heterogeneous tissue oxygenation: renal ischemia/reperfusion injury in a large animal,” J. Biomed. Opt. 18(3), 035001 (2003). JBOPFO 1083-3668 10.1117/1.JBO.18.3.035001 PMC4023644 23456040

[9] Chen X. et al., “In vivo real-time imaging of cutaneous hemoglobin concentration, oxygen saturation, scattering properties, melanin content, and epidermal thickness with visible spatially modulated light,” Biomed. Opt. Express 8(12), 5468–5482 (2017). BOEICL 2156-7085 10.1364/BOE.8.005468 29296481 PMC5745096

[10] Yaroslavsky A. et al., “Delineating nonmelanoma skin cancer margins using terahertz and optical imaging,” J. Biomed. Photonics Eng. 3(1), 010301 (2017). 10.18287/JBPE17.03.010301

[11] Rohrbach D. J. et al., “Preoperative mapping of nonmelanoma skin cancer using spatial frequency domain and ultrasound imaging,” Acad. Radiol. 21(2), 263–270 (2014). 10.1016/j.acra.2013.11.013 24439339 PMC3960981

[12] Saager R. B. et al., “A light emitting diode (LED) based spatial frequency domain imaging system for optimization of photodynamic therapy of nonmelanoma skin cancer: quantitative reflectance imaging,” Lasers Surg. Med. 45(4), 207–215 (2013). LSMEDI 0196-8092 10.1002/lsm.22139 23619900 PMC3940278

[13] Rohrbach D. J. et al., “Characterization of nonmelanoma skin cancer for light therapy using spatial frequency domain imaging,” Biomed. Opt. Express 6(5), 1761–1766 (2015). BOEICL 2156-7085 10.1364/BOE.6.001761 26137378 PMC4467704

[14] Ponticorvo A. et al., “Evaluating clinical observation versus spatial frequency domain imaging (SFDI), laser speckle imaging (LSI) and thermal imaging for the assessment of burn depth,” Burns 45(2), 450–460 (2019). BURND8 0305-4179 10.1016/j.burns.2018.09.026 30327232 PMC6420831

[15] Zeng B. et al., “Handheld spatial frequency domain imager for noninvasive Sjögren’s syndrome labial salivary gland biopsy,” Biomed. Opt. Express 12(8), 5057–5072 (2021). BOEICL 2156-7085 10.1364/BOE.426683 34513242 PMC8407847

[16] Cuccia D. J. et al., “Quantitation and mapping of tissue optical properties using modulated imaging,” J. Biomed. Opt. 14(2), 024012 (2009). JBOPFO 1083-3668 10.1117/1.3088140 19405742 PMC2868524

[17] Saager R. B. et al., “Method for depth-resolved quantitation of optical properties in layered media using spatially modulated quantitative spectroscopy,” J. Biomed. Opt. 16(7), 077002 (2011). JBOPFO 1083-3668 10.1117/1.3597621 21806282 PMC3146548

[18] O’Sullivan T. D. et al., “Diffuse optical imaging using spatially and temporally modulated light,” J. Biomed. Opt. 17(7), 0713111 (2012). JBOPFO 1083-3668 10.1117/1.JBO.17.7.071311 PMC3607494 22894472

[19] Tseng S. H. et al., “Determination of optical properties of superficial volumes of layered tissue phantoms,” IEEE Trans. Biomed. Eng. 55(1), 335–339 (2008). IEBEAX 0018-9294 10.1109/TBME.2007.910685 18232377 PMC2629128

[20] Majedy M. et al., “A melanoma cancer screening framework based on depth-resolved light scattering,” SPIE Proc. PC12352, PC1235205 (2023). JBOPFO 1083-3668 10.1117/12.2649645

[21] Schotland J. C. Haselgrove J. C. Leigh J. S., “Photon hitting density,” Appl. Opt. 32(4), 448–453 (1993). APOPAI 0003-6935 10.1364/AO.32.000448 20802710

[22] Cuccia D. J. et al., “Modulated imaging: quantitative analysis and tomography of turbid media in the spatial-frequency domain,” Opt. Lett. 30(11), 1354 (2005). OPLEDP 0146-9592 10.1364/OL.30.001354 15981531

[23] Farrell T. J. Patterson M. S. Wilson B., “A diffusion theory model of spatially resolved, steady-state diffuse reflectance for the noninvasive determination of tissue optical properties in vivo,” Med. Phys. 19(4), 879–888 (1992). MPHYA6 0094-2405 10.1118/1.596777 1518476

[r24] Seo I. S. Hayakawa C. K. Venugopalan V., “Radiative transport in the delta-P1 approximation for semi-infinite turbid media,” Med. Phys. 35(2), 681 (2008). MPHYA6 0094-2405 10.1118/1.2828184 18383690 PMC3509770

[25] Horan S. T. et al., “Recovery of layered tissue optical properties from spatial frequency-domain spectroscopy and a deterministic radiative transport solver,” J. Biomed. Opt. 24(7), 071607 (2019). JBOPFO 1083-3668 10.1117/1.JBO.24.7.071607 PMC6995875 30456934

[r26] Farina B. et al., “Monte Carlo simulation of light fluence in tissue in a cylindrical diffusing fibre geometry,” Phys. Med. Biol. 44(1), 1–11 (1999). PHMBA7 0031-9155 10.1088/0031-9155/44/1/002 10071871

[27] Sandell J. L. Zhu T., “Monte Carlo simulation of light fluence calculation during pleural PDT,” Proc. SPIE 8568, 85680U (2013). PSISDG 0277-786X 10.1117/12.2005944 PMC4437723 25999640

[28] Piessens R., “The Hankel transform,” in The Transforms and Applications Handbook, Poularikas A. D., Ed., 2nd ed., CRC Press LLC (2000).

[29] Carp S. A. Prahl S. A. Venugopalan V., “Radiative transport in the delta-P1 approximation: accuracy of fluence rate and optical penetration depth predictions in turbid semi-infinite media,” J. Biomed. Opt. 9(3), 632–647 (2004). JBOPFO 1083-3668 10.1117/1.1695412 15189103

[30] Seo I. S., “Diffuse reflectance spectroscopy for epithelial tissues,” University of California, Irvine (2007).

[31] Saager R. B. et al., “Multilayer silicone phantoms for the evaluation of quantitative optical techniques in skin imaging,” Proc. SPIE 7567, 756706 (2010). PSISDG 0277-786X 10.1117/12.842249

[32] Pogue B. W. Patterson M. S., “Review of tissue simulating phantoms for optical spectroscopy, imaging and dosimetry,” J. Biomed. Opt. 11(4), 041102 (2006). JBOPFO 1083-3668 10.1117/1.2335429 16965130

[33] Saager R. B. et al., “Low-cost tissue simulating phantoms with adjustable wavelength-dependent scattering properties in the visible and infrared ranges,” J. Biomed. Opt. 21(6), 067001 (2016). JBOPFO 1083-3668 10.1117/1.JBO.21.6.067001 27292135 PMC4904063

[34] Belcastro L. et al., “Handheld multispectral imager for quantitative skin assessment in low-resource settings,” J. Biomed. Opt. 25(8), 082702 (2020). JBOPFO 1083-3668 10.1117/1.JBO.25.8.082702 32755076 PMC7399474

[35] Saager R. B. et al., “Portable (handheld) clinical device for quantitative spectroscopy of skin, utilizing spatial frequency domain reflectance techniques,” Rev. Sci. Instrum. 88(9), 94302 (2017). RSINAK 0034-6748 10.1063/1.5001075 PMC5589466 28964218

[36] Horan S. T., “Spectral methods for solving the radiative transport equation in single and double spherical harmonics and their application to optical imaging,” UC Irvine Electronic Theses and Dissertations (2020).

[37] Belcastro L. et al., “Beneath the skin: multi-frequency SFDI to detect thin layers of skin using light scattering,” Proc. SPIE 12352, 1235209 (2023). PSISDG 0277-786X 10.1117/12.2648545

